# Adequacy of Existing Surveillance Systems to Monitor Racism, Social Stigma and COVID Inequities: A Detailed Assessment and Recommendations

**DOI:** 10.3390/ijerph182413099

**Published:** 2021-12-12

**Authors:** Chandra L. Ford, Bita Amani, Nina T. Harawa, Randall Akee, Gilbert C. Gee, Majid Sarrafzadeh, Consuela Abotsi-Kowu, Shayan Fazeli, Cindy Le, Ezinne Nwankwo, Davina Zamanzadeh, Anaelia Ovalle, Monica L. Ponder

**Affiliations:** 1 Center for the Study of Racism, Social Justice & Health, Department of Community Health Sciences, Fielding School of Public Health, University of California at Los Angeles, Los Angeles, CA 90095, USA; Bitaamani@CDrewU.edu (B.A.); nharawa@mednet.ucla.edu (N.T.H.); rakee@ucla.edu (R.A.); gilgee@ucla.edu (G.C.G.); consuela.abotsi@gmail.com (C.A.-K.); lecin@g.ucla.edu (C.L.); enwankwo@ucla.edu (E.N.); 2 Department of Urban Public Health, College of Science & Health, Charles R. Drew University of Medicine & Science, Los Angeles, CA 90059, USA; 3 Department of Medicine, Division of General Internal Medicine and Health Services Research, Los Angeles, CA 90095, USA; 4 Department of Public Policy, Luskin School of Public Affairs, University of California at Los Angeles, Los Angeles, CA 90095, USA; 5 Department of Computer Science, University of California at Los Angeles, Los Angeles, CA 90095, USA; majid@cs.ucla.edu (M.S.); shayan.fazeli@gmail.com (S.F.); davina@cs.ucla.edu (D.Z.); anaeliaovalle@g.ucla.edu (A.O.); 6 Department of Communication, Culture & Media Studies, Cathy Hughes School of Communication, Howard University, Washington, DC 20059, USA; monica.ponder@Howard.edu

**Keywords:** big data, surveillance, racism, evaluation, stigma, pandemic

## Abstract

The populations impacted most by COVID are also impacted by racism and related social stigma; however, traditional surveillance tools may not capture the intersectionality of these relationships. We conducted a detailed assessment of diverse surveillance systems and databases to identify characteristics, constraints and best practices that might inform the development of a novel COVID surveillance system that achieves these aims. We used subject area expertise, an expert panel and CDC guidance to generate an initial list of N > 50 existing surveillance systems as of 29 October 2020, and systematically excluded those not advancing the project aims. This yielded a final reduced group (*n* = 10) of COVID surveillance systems (*n* = 3), other public health systems (4) and systems tracking racism and/or social stigma (*n* = 3, which we evaluated by using CDC evaluation criteria and Critical Race Theory. Overall, the most important contribution of COVID-19 surveillance systems is their real-time (e.g., daily) or near-real-time (e.g., weekly) reporting; however, they are severely constrained by the lack of complete data on race/ethnicity, making it difficult to monitor racial/ethnic inequities. Other public health systems have validated measures of psychosocial and behavioral factors and some racism or stigma-related factors but lack the timeliness needed in a pandemic. Systems that monitor racism report historical data on, for instance, hate crimes, but do not capture current patterns, and it is unclear how representativeness the findings are. Though existing surveillance systems offer important strengths for monitoring health conditions or racism and related stigma, new surveillance strategies are needed to monitor their intersecting relationships more rigorously.

## 1. Introduction

The populations impacted most by COVID are also impacted by racism and related social stigma; as the COVID pandemic is revealing, however, traditional surveillance tools do not capture the myriad of ways racism and social stigma contribute to health inequities. Racism has been defined as “a system of structuring opportunity and assigning value based on the social interpretation of how one looks (which is what [people] call ‘race’), that unfairly disadvantages some individuals and communities, unfairly advantages other individuals and communities, and saps the strength of the whole society through the waste of human resources” [1]. COVID surveillance systems provide timely overall data on diagnoses, deaths and other outcomes (e.g., hospitalizations); however, they often lack detailed information by race/ethnicity, which is necessary to identify disparities, and almost never include indicators of racism and other root causes. The failure to capture and report data by race/ethnicity obscures disparities and the structural determinants that undergird them [2,3]. The first step toward addressing the root causes of these public health problems is generating data about them [3]. Documenting racism is necessary to generate valid estimates of effect, attribute appropriate causal contributions to racism and identify targets for policy interventions. Examples of the kinds of data such a system should include are provided in Box 1.

Box 1 **Three examples of racism indicators.**

- **Hate Crimes**, which have increased among Asian populations, in particular, during the COVID pandemic. Annual reports are issued by the US Federal Bureau of Investigations (FBI) Hate Crimes Division. They summarize racist targeting of individuals and serve as an indicator of racialized social stigma.
- **Housing Discrimination**, which may occur on the basis of race, ethnicity or other characteristics, can increase social vulnerability and exacerbate adherence with COVID guidelines. National data are available from Home Mortgage Disclosure Act reports.
- **Residential segregation**, which is a key measure of structural racism and estimates the nature and pervasiveness of social exclusion that Black, Indigenous and People of Color groups experience. The indices available via the US Census provide objective data on the amount of racial exclusion in a place, which affects access to care and exposure to hazards.

Lessons learned from prior epidemics, such as the HIV epidemic, include that stigma exacerbates disease-mitigation efforts among the most vulnerable populations, contributes to mistrust of public health messages, delays accessing recommended services and reduces adherence to prescribed treatment regimens [4,5,6]. Anecdotal and empirical evidence exists of COVID-19-related stigma [7] and related violence against Asians and members of other groups [8,9]. Traditionally, public health surveillance is used to monitor trends in environmental conditions, disease outcomes and/or risk factors; identify hotspots where disease and/or risk are concentrated; and predict potential threats to the health of the public early in order to intervene on them. Efforts to mitigate the root causes of COVID-19 inequities among diverse vulnerable populations could be improved by developing new surveillance tools that capture the intersecting ways racism, stigma and disease co-occur; however, we are aware of no surveillance systems that do so.

We address this gap by conducting detailed assessments of existing surveillance systems and databases to identify key methods, characteristics and best practices that might inform the development of a surveillance system that is rooted in anti-racism and equity, which former American Public Health Association president, Camara Jones, defines as “assurance of the conditions for optimal health for all people” [1]. Because it is not possible to identify every system that has existed or that currently exists, this review highlights the potential contributions and constraints of several categories of systems. Based on the results of the review, we make recommendations for developing the type of novel surveillance system needed to monitor the intersecting pandemics of racism, stigma and COVID-19.

## 2. Materials and Methods

### 2.1. Research Purpose and Hypotheses

The purpose of this research was to identify characteristics of existing surveillance systems that might inform the development of a novel COVID monitoring system that tracks COVID-19-outcomes in real time, as well as key forms of stigma and racism that affect them. This detailed assessment identifies existing surveillance and monitoring systems that collectively might support the aims of Project REFOCUS. Three questions anchor our approach (Box 1). First, how can Project REFOCUS remain attentive to ways that mitigation and containment efforts can harm racialized communities (e.g., contract tracing of undocumented persons)? Secondly, can a crisis response to inequities challenge debunked ideas about race that are being regenerated and reformulated in the early 21st century (e.g., in the discourse on the causes of disparities in the COVID-19 pandemic)? Finally, what alternative strategies to monitoring this public health problem might avoid the use of surveillance strategies known to harm Black and Brown communities?

### 2.2. Methods

A multidisciplinary team of epidemiologists, community health scientists, critical race theorists, policy experts, computer scientists and data scientists conducted a baseline rapid environmental scan of existing surveillance systems on which a future COVID surveillance system to monitor the structural drivers of racial/ethnic inequities might build. To determine the scope of the environmental scan, we established a panel of diverse surveillance experts (i.e., expert panel) and solicited input from the panel and from experts at the Centers for Disease Control and Prevention (CDC) on the types of information needed to support the national pandemic response. For example, the CDC shared information about data sources being developed by partners, and it emphasized the need for data used for the COVID response to be available in as near real time as possible. We used subject area expertise, input from the advisory panel of experts on surveillance and CDC consultations to conduct a baseline environmental scan, which yielded an initial list of N > 50 surveillance systems to include, as of 29 October 2020, and systematically excluded systems that were less informative to the project aims. This list included COVID-19 surveillance systems (*n* = 30), other public-health surveillance systems (*n* = 12) and systems (*n* = 8) that track racist incidents. The final reduced group (*n* = 10) of COVID surveillance systems (*n* = 3), other public-health surveillance systems (4) and systems tracking incidents of racism and/or social stigma (3) was evaluated based on two sets of criteria: one was a standard set of criteria CDC uses to evaluate surveillance systems, and the other was a set of additional criteria that we generated based on the principles of Public Health Critical Race Praxis (PHCRP)/Critical Race Theory (CRT) [10]. Critical Race Theory originated in law as an anti-racism movement seeking to explain the ways in which racism continues to be embedded in jurisprudence in the post-civil-rights era. PHCRP extends CRT to enable the systematic integration of anti-racism approaches into health-equity research [11,12]. In this project, PHCRP/CRT informs the conceptualizations of racism and social stigma and encourages a racially conscious assessment of the systems. For instance, it explains why the failure to collect needed data on race, ethnicity and racism reinforces White supremacy.

Table 1 lists the two sets of criteria used to conduct the detailed assessment and the key question each criterion addresses. The first five criteria—usefulness, timeliness, flexibility, simplicity and data quality—are standard criteria by which CDC and others evaluate surveillance systems. The second set of criteria focuses on the intersectionality of COVID-19 and racism [13,14,15]. They assess whether a system has data on race/ethnicity, which is needed to identify disparities and document progress toward equity; measures of racism, which are needed to identify the underlying drivers of inequities; and measures of stigma [16].

## 3. Results

### 3.1. COVID-19 Surveillance Systems

Table 2 presents the COVID surveillance systems (*n* = 12) by the COVID-19 outcomes they include; the frequency with which data are updated; and whether the system includes data on race/ethnicity, stigma and racism. An “x” indicates that the system has the specified characteristic or type of data. These systems obtain data on the COVID outcomes either directly from state and local health departments or via other agencies (e.g., CDC) or databases (e.g., 1.3 acres) who obtain it from health departments. The systems vary considerably in which metrics they include. The most reliably reported COVID-19 data are on diagnoses and deaths; the inclusion of other factors (e.g., hospitalizations), however, could provide earlier signals of potential disparities due to, for example, limited access to hospital beds (Table 2).

As many COVID-19 surveillance systems report similar types of information (e.g., overall diagnosis rates), we purposively present a representative system for each of three different types of these systems to understand the unique insights each might offer: the COVID-19 Dashboard at Johns Hopkins University, the COVID Tracking Project at *The Atlantic* magazine and the Olivia prototype at UCLA.

**Figure 1:**
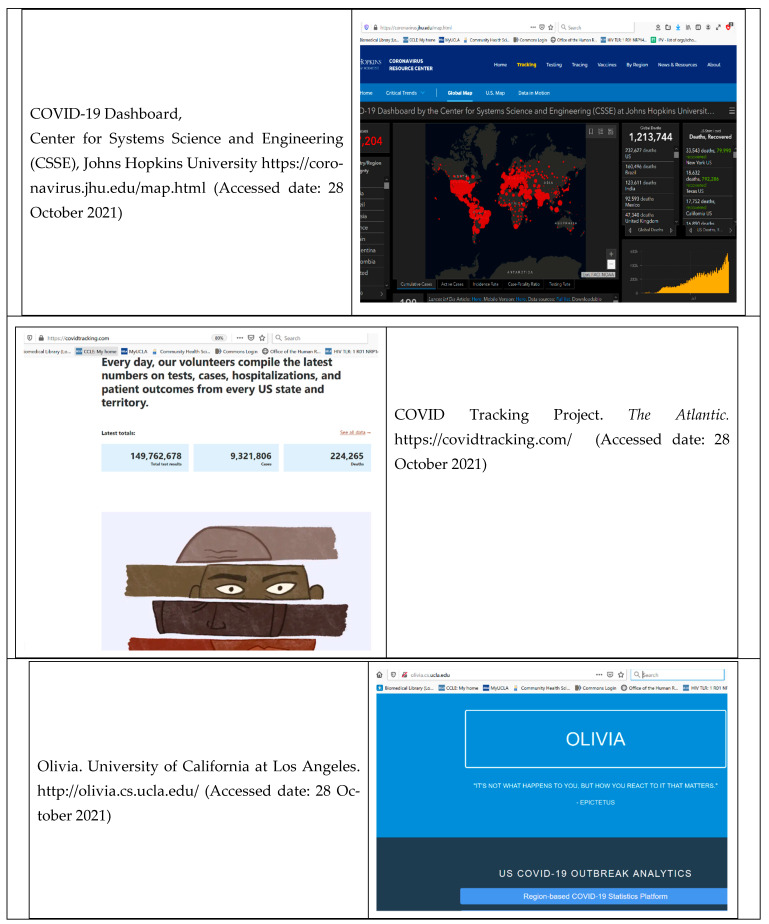
Selected COVID surveillance systems.

The Johns Hopkins Center for Systems Science and Engineering (CSSE) system (Figure 1) was among the first COVID surveillance systems to be developed. Its epidemiologic value stems from its usefulness, timeliness and comprehensiveness as a resource for tracking existing and emerging COVID-19 trends; and the high level of detailed information the dashboard provides about the data it presents. This information is provided in a manner that is accessible to different types of end-users, ranging from epidemiologists to the media and general public. Additional strengths include the ease with which these diverse end-users can access and query the system to visualize a wide range of COVID outcomes, the clarity and high quality of the data visualizations, and the availability of extensive sets of documentation explaining this system and its limitations. Similar to most other COVID systems, it has limited data on race and ethnicity and no data on racism or stigma; however, recently it has begun to document states with race/ethnicity data and include some healthcare indicators. Both the detailed documentation it provides about its data sources and the care taken to ensure transparency bolster confidence in the quality of these data. Though the level of complexity involved in maintaining the system was not evaluated, the number of visualizations that can be queried and the efficiency with which the dashboard can be searched suggest a high level of complexity undergirds the system.

Until its retirement in March 2021, the COVID Tracking Project (CTP) (Figure 1) was another leading resource for monitoring COVID. Its simple, informative interface made it easy for the media, the general public and epidemiologists to use. What most distinguishes it from other COVID systems, including the JHU COVID-19 Dashboard, is its inclusion of COVID data by race/ethnicity wherever possible though the JHU dashboard has recently begun to indicate which states track race/ethnicity data. The data were updated daily, thus enhancing the usefulness and timeliness of this system. The data primarily come from health departments; therefore, they are subject to the same critiques as those made of health department data more broadly: they are often incomplete, especially regarding race/ethnicity variables. Nevertheless, CTP stands out among COVID surveillance systems because it provides the best available data on race, ethnicity and disparities; moreover, it explains data constraints related to race and ethnicity very transparently. The level of detail with which the documentation describes the race/ethnicity data bolsters overall confidence in the quality of the data on which the dashboard relies. While the inclusion of race and ethnicity data enable monitoring of racial/ethnic disparities, CTP did not attempt to capture measures of racism or other indicators of their root causes.

Olivia (Figure 1) is a protocol currently under development at UCLA. It draws its data from other COVID surveillance systems (e.g., 1.3 acres) and from social and economic sources, such as the census; therefore, it is subject to the same limitations and strengths as those of the underlying data sources. With respect to monitoring the co-occurrence of racism, social stigma and COVID, its key strengths include (1) its focus on mapping links between social inequalities and health inequities, and (2) its status as “under development”. Because it is still under development and focused on health inequities, Olivia provides an opportunity to embed equity and anti-racism approaches in the system’s architecture, which is ideal for the development of a novel system. This can also be helpful because information needs change over the course of the pandemic. Olivia’s relatively small size gives it the flexibility needed to adjust as the pandemic evolves.

A growing number of COVID-19 systems now exist. Collectively, they help monitor infectious disease and, to some degree, racial/ethnic inequities, but they do not generally monitor social inequities, such as racism (Figure 1). The JHU CSSE system is among the best system for monitoring overall COVID trends, and it is already widely used by the public, researchers, policymakers, the media and public health professionals; however, it lacks good data on race/ethnicity, racism and related stigma. Moreover, there is no evidence of an equity or anti-racism orientation. Until March 2021, the CTP dashboard provided the best national data by race/ethnicity on COVID diagnoses and deaths, but it did not contain data on specific stigma- or racism-related indicators. Overall, the dashboard relies on data primarily from state and local health departments, which vary considerably in how adequately they collect race/ethnicity data, the amount of data missing for specific indicators and the level of detail in the documentation provided about the data. Although the infrastructure of the CTP may serve as a useful model, unfortunately, the dashboard is no longer being updated. The Olivia prototype draws on many of the same data sources as other COVID-19 systems do; however, its expressed focus on inequities at such an early stage of its development makes it a compelling option for supporting the development of a novel system from scratch that focuses on the co-occurrence of racism, stigma and COVID inequities.

### 3.2. Other Public-Health Surveillance Systems

**Figure 2:**
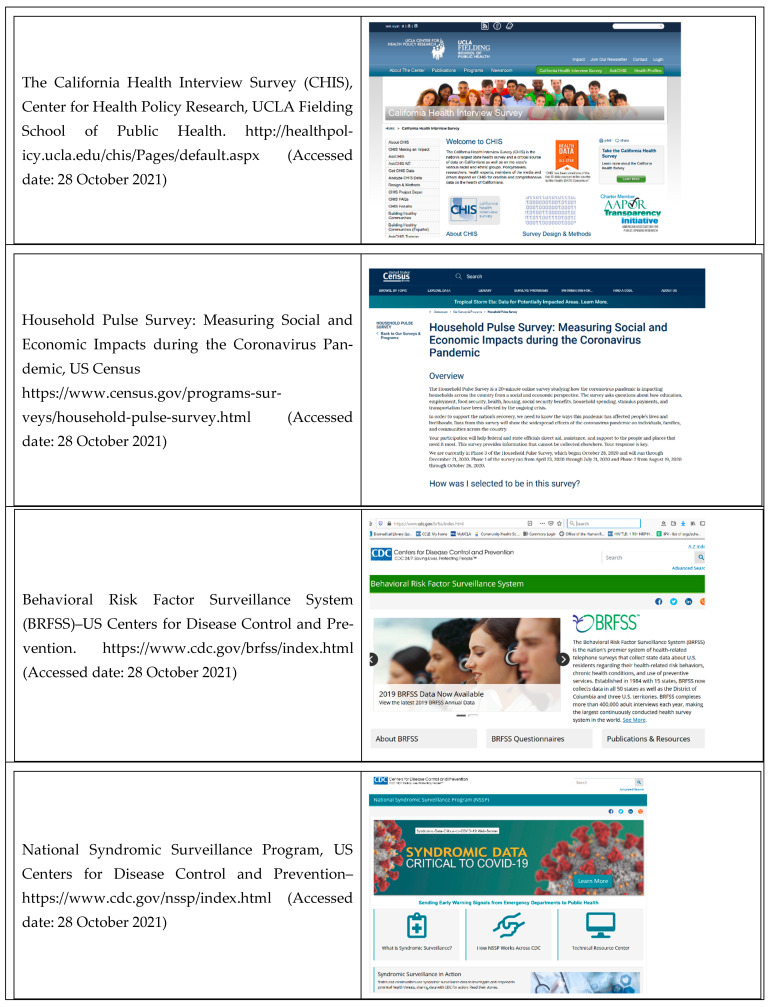
Other public-health surveillance systems.

Many of the approaches and metrics used in traditional public-health surveillance systems complement those of the flexible real-time COVID-19 systems. Table 3 lists those (*n* = 4) for which detailed assessments were conducted based on whether each system includes specified race/ethnicity data, measures of stigma and racism; different COVID-19 metrics; and the frequency with which the data are updated. An “x” indicates that a system has the specified data or characteristic. From this list, one statewide system, the California Health Interview Survey, and three national ones—the Behavioral Risk Factor Surveillance System, the US Census Household Pulse and CDC National Syndromic Surveillance Program (NSSP)—were identified as offering particularly useful insights for developing the novel system. Each of these was designed for routine administration of population surveys assessing a variety of factors, including the social determinants of health, healthcare outcomes (e.g., care-seeking) and, in some cases, stigma, race/ethnicity and racism (Figure 2).

According to its website, CHIS is the nation’s largest statewide annual survey. Researchers, policymakers and others use it to assess the health of California residents and their access to and use of healthcare services. It scores high among both the traditional public-health surveillance criteria and the additional equity-related criteria we specified. CHIS is a publicly available resource. Its usefulness is bolstered by its user-friendly interface. Its relevance stems, in part, from the oversampling of the many diverse racial/ethnic populations that reside in California, including Asians and Pacific Islanders (APIs), making it an excellent resource for tracking the pandemic among API residents who have been impacted disproportionately by COVID [17]. To address this issue more directly, CHIS has begun collecting some data more frequently, and this improves its timeliness and moves it slightly closer toward the real-time monitoring of outcomes. In addition to the publicly available data, end-users can apply for access to the private data, which contain more detailed information on the respondents, including some personal health information. Despite its strengths, CHIS has two significant weaknesses with respect to a novel COVID surveillance system. Because it is limited to California, data on other states are not available. However valid its measures, protocols and findings are for California, they may not be generalizable to other regions. Secondly, conducting this annual survey is a large undertaking. It is unclear whether it can fully capture and share information as rapidly as is needed to respond to an infectious-disease pandemic.

The US Census Household Pulse Survey conducts quick weekly to bi-weekly assessments of households to understand how the COVID-19 pandemic is affecting the physical and mental health of US residents and to assess certain social determinants of health. It is a national survey that began in April 2020, with sampling at the national, state and metropolitan statistical areas (for 15 MSAs). The Census infrastructure enables linkage to the social and economic datasets available via the census and may help sustain the survey. The ability to link the survey data on psychosocial factors or mental health to census-designated places that are more granular than the county- or state-level data available in most COVID systems is another strength. Though sample sizes exceed N = 39,000/week, the response rates may be low. Of concern, the populations who are most likely to experience racism and social stigma may be the very populations the sampling strategy misses. Perhaps its greatest weakness, however, is the irregularity with which it is administered, which generates information bias and makes it difficult to document trends over time.

The CDC Behavioral Risk Factor Surveillance System (BRFSS) is among the nation’s oldest and most widely used public-health surveillance systems for monitoring trends in psychosocial factors, health behaviors and healthcare utilization [18]. States use its modules to conduct annual assessments of the health behaviors, health and healthcare of state residents. A notable strength of BRFSS is that it includes many social determinants of health and demographic factors, including race and ethnicity, which enable the estimation of racial/ethnic disparities [18]. Furthermore, many measures of psychosocial factors are based on psychometrically validated instruments. In addition, the BRFSS’s well-established survey protocols may improve data quality (e.g., completion rates) as compared to “one off” surveys [18]. These strengths are hampered, however, by the time and considerable effort required to implement the survey each year. BRFSS cannot meet the information needs of the rapidly evolving COVID-19 pandemic unless it increases the frequency of data collection and reporting, and decrease the time from data collection to reporting. To reach the diverse populations impacted most by racism and stigma, it may also need to revise its sampling strategy, which includes sampling from both landlines and cell phones; however, sampling based on landlines may induce bias due to the limited inclusion of marginalized populations.

The National Syndromic Surveillance Program (NSSP) is a model of innovation and efficiency with respect to its ability to relay in real-time information collected as people present to emergency departments. Its flexibility and timeliness in sharing information are its greatest strengths. These strengths are possible because it relies in part on mobile and other technology. Despite these unique strengths, its usefulness for monitoring the co-occurrence of racism and COVID inequities is severely constrained by two data-quality concerns. First, as our expert panel advised, the race/ethnicity data submitted by NSSP shares are often incomplete, making it difficult to track racial/ethnic inequities and reducing the precision of estimates generated based on the data. Secondly, the program does not systematically collect any information on patients’ experiences with racism and related social stigma. Fortunately, recommendations for collecting data on race/ethnicity, racism and other social exposures (e.g., stigma) in clinical settings have recently been published, suggesting that they could be added to this system [19,20].

Overall, the systems in this category typically include measures of race/ethnicity, and some include measures of racism and/or stigma; however, they generally lack the real-time timeliness that characterizes COVID surveillance systems. That level of timeliness is necessary for a surveillance tool to be able to inform responses to a rapidly evolving pandemic. One exception to this is the NSSP, which is a model of timeliness; however, it is weakened by incomplete records and missing data on race/ethnicity, thus affecting the data quality and the utility of the system. In addition, NSSP data are based on individuals presenting for care; it does not systematically collect data from the most socially vulnerable populations in communities, including those who avoid or delay care-seeking due to perceived stigmatization or racism.

### 3.3. Systems Tracking Racism and Related Social Stigma

The final category of systems we examined was those that monitor racism and related forms of social stigma (Table 4). In general, these systems retroactively present reported incidents of discrimination that were directed at individuals on the basis of their presumed or actual status as a member of a protected class based on race, ethnicity, sexual orientation, etc. The data are made available at the state level, but, in some instances, they are also available for other geographies, such as the county or metropolitan statistical area, too, depending in part on what local initiatives exist to track the information. The systems typically provide annual summaries of incidents occurring during the prior year; therefore, they do not currently provide the assessments in real time. How useful the historical data are for predicting future incidents or trends has not yet been established. Table 4 shows the reduced list of these systems by COVID-19 outcomes, race/ethnicity categories, measures of stigma and racism and the frequency with which each system is updated. An “x” indicates that a system includes the specified data or characteristic. In general, these systems have relatively few observations per year or region, reflecting the stringent criteria they use to operationalize racism (e.g., the perpetrator must have explicitly made known their racist intent). This suggests the reported values underestimate the true level of racism exposures, because they only account for incidents that are perceived as racist and then reported [21]. In addition, information on how specific types of data were obtained and other factors related to data quality are not always as detailed in the documentation as that provided in epidemiologic surveillance systems. They vary in how real time they are; generally, they are less timely than COVID-19 surveillance systems, but timelier than many traditional public health systems. Because they are longstanding, well-respected systems that target problems few other surveillance programs address, however, the systems capture best practices for documenting overt forms of interpersonal discrimination and inferred stigma. These measures complement the types of stigma and racism measures that might be generated from analyses of social media data (e.g., Twitter), which has become an increasingly popular platform on which to monitor racial stigma [8]. We highlight quite different approaches to monitoring racism and social stigma: that of the FBI’s hate-crime-reporting division, Project Implicit and Stop AAPI Hate.

The US Federal Bureau of Investigation’s (FBI’s) Uniform Crime Reporting (UCR) Program is the authoritative federal agency on hate incidents and hate crimes targeting people on the basis of race, ethnicity, religion, sexual orientation or gender identity and other characteristics. The FBI collects these reports directly from states and elsewhere, and generates summary statistics of the patterns annually. Hate incidents on the basis of race or other protected classes constitute interpersonal forms of racism; they also serve as an informal indicator of a group’s stigmatized status, though this use has not yet been validated for public-health surveillance [4,22,23]. As previously suggested, these systems are not designed to capture structural and other mechanisms that are difficult for individuals to perceive. Moreover, even the reports of racism may be undercounts, as people who experience racism may not report it [22]. Limitations of these data include that, while the documentation defines each type of hate incident, the methods that each state uses to collect and report the data are not known. In addition, delays of a year or longer between data collection and reporting are common. This lag in timeliness can render the data less useful for understanding present trends.

**Figure 3:**
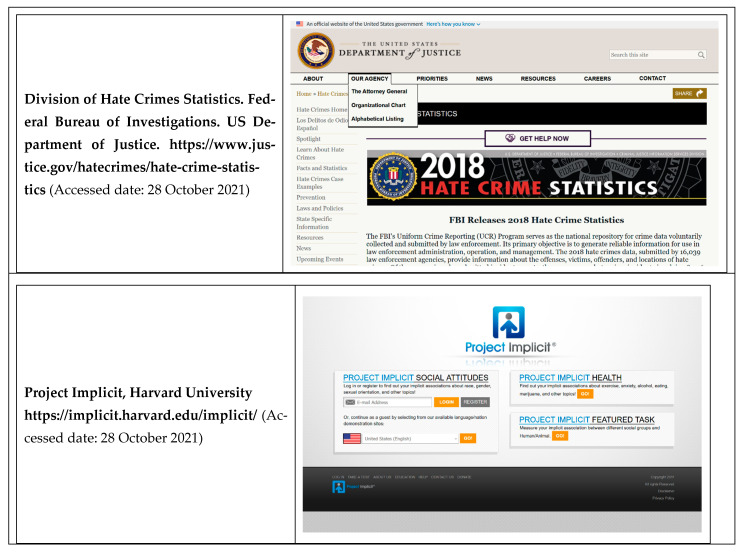

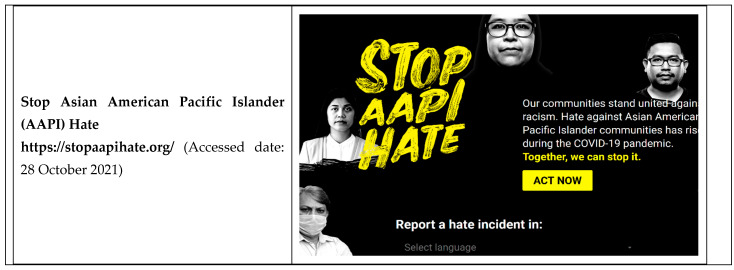
Selected racism-monitoring systems.

We are aware of no system that routinely tracks distributions of implicit racial bias across the nation. Project Implicit (Figure 3) pioneered the assessment of such biases by using an online tool, and the results may serve as proxies indicating stigmata linked to a group [23], because the instrument is designed to reveal groups against which a user subconsciously holds negative sentiments.

Individuals self-administer the electronic instrument, which measures the ease with which they associate images of, for instance, White vs. Black people with positive vs. negative characteristics, respectively [23,24]. Simplicity characterizes the user experience. Educators, researchers and the general public use this tool, though questions have been raised about its validity in certain circumstances. To our knowledge, no COVID surveillance system incorporates it as a metric to systematically capture racial biases or stigma in real time among populations. To be useful in a COVID monitoring system would require the ability to deploy the instrument rapidly or to integrate population-based data previously collected from it into the novel surveillance system.

Stop AAPI Hate was a community-originated initiative in response to evidence that Asians were being scapegoated for COVID and targeted for hate crimes based on racialized stigma. Several characteristics distinguish this system from others. First, it is a population-specific tool; its design and the outreach efforts used to connect people focus specifically on Asians and Pacific Islanders. The system seeks to document their experiences with interpersonal violence and discrimination, which might otherwise be missed or undercounted by public health officials and policymakers. Second, it collects narrative data (i.e., stories) based on reports individuals submit about experiences they have had. Individuals can submit reports on an ongoing basis, but the frequency with which the data are analyzed and made available to the public is not known. While the system provides rich insights to characterize experiences with hate incidents and hate crimes against Asian people during the pandemic, it does not systematically collect data on COVID-related outcomes (e.g., COVID deaths). Nor is it possible to establish the generalizability or representativeness of the data as population denominators are not known and individuals may report anonymously. Nevertheless, its use of qualitative data to document these exposures and characterize their impacts in this underserved set of populations offers an important alternative way to conduct surveillance on the intersections of racism, related social stigma and COVID.

In developing a novel integrated surveillance system that can monitor racism, racialized social stigma and COVID outcomes in real time, much can be learned from systems that have been monitoring racism over the decades and more recently. Each of the resources highlighted here is free and publicly available, and they each provide unique information that is difficult to find elsewhere. There are two overall limitations, however. First, the data they report reflect historical occurrences; they generally do not indicate real time patterns. Secondly, few such systems account for health outcomes [25].

## 4. Discussion

### 4.1. Overall Discussion

This assessment identified ways in which three types of surveillance systems can inform the development of a novel PHCRP/CRT-informed COVID surveillance system. The greatest potential contribution of COVID-19 surveillance systems is their use of data in real-time (e.g., daily) or near real time (e.g., weekly). They routinely include data on COVID-19 diagnoses and deaths, though more systematic inclusion of data on hospitalizations and other outcomes (case positivity ratio, percentage vaccinations distributed and administered) would better enable prediction of the potential for disparities to emerge.

No consistent set of metrics is used to capture COVID and other outcomes. Rarely do they contain complete data by race/ethnicity, thus making it difficult to track racial/ethnic inequities. To standardize the measurement and reporting of COVID-19 indicators and racism-related measures would enhance the ability to monitor and compare trends over time, regions and populations. Missing data for some regions or places affect the ability to monitor those specific places (e.g., counties), as well as the broader places (e.g., state) of which they are a part. This information gap could be resolved if all comparable places (e.g., counties) for a specified geography (e.g., state) used standard metrics. These systems rely heavily on health-department data. The resources that health departments across the nation have at their disposal to address COVID-19 vary considerably. Some health departments have a lot of resources that can be dedicated to COVID, while others have very few. To support a national response, resources may need to be provided to those health departments that lack resources in order to support them in developing comprehensive systems for monitoring the co-occurrence of COVID inequities and social justice crises.

With respect to other public-health disease-surveillance systems, a myriad of strengths and weaknesses exist that largely complement those of COVID surveillance systems. Many of the psychosocial factors (e.g., perceived discrimination) obtained via surveys or polls are valid, reliable measures that improve generalizability. In addition to the public version of the data, some (e.g., CHIS) also have private versions that have more detailed information and personal identifiers. Users can apply to access these data to conduct research. Despite these strengths, these systems lack the timeliness that characterizes COVID surveillance systems, and they do not fully capture the intersectionality of racism, COVID and stigma.

The type of information available in systems that track racism (e.g., hate crimes) is difficult to find elsewhere. The FBI hate-crimes databases are the authoritative source of information on hate crimes and hate incidents. Because they focus on the most extreme forms of interpersonal racism and rely on people reporting incidents, however, these reports likely undercount the true number of hate incidents occurring each year. Furthermore, as Ford suggests, certain groups may be less likely than others either to report racist incidents or to attribute the experiences to race [22,26]. The focus on overt hate crimes also inherently underestimates implicit forms of racism and structural racism, which is known to drive health inequities [22].

Emerging technologies and analytic techniques such as machine learning, artificial intelligence and social media analyses (e.g., Twitter and Facebook) provide opportunities to generate powerful insights about health and social phenomena; however, racial and other biases embed them in ways that have yet to be fully understood [27,28]. The use of PHCRP approaches [29] and the reliance on a pandemic ethics dashboard [30] are important first places to start to address these issues. A growing number of social movements, such as Data for Black Lives (D4BL), challenge practices that direct state surveillance efforts at racial/ethnic minority communities [16,31]. They seek to limit surveillance in these communities, educate communities about the ways that surveillance operates in communities and expand community access to the tools of data science for autonomous use by those whom these systems have historically targeted. In particular, law enforcement systems disproportionately target Black and Brown communities. Addressing these concerns is necessary for the system to adhere to principles of equity and antiracism. Information shared with them from public-health surveillance systems about individuals and communities may harm them.

Surveillance systems can also consider linkages to “crowdsourced” data, referring to the general idea that laypersons (who are not trained researchers per se) collect information that are shared into a common database. Some examples are crowdsourced data from residents to gather data regarding geospatial maps [32], security and sexual violence [33] and food access [34]. Crowdsourced data have the potential to supplement official surveillance systems, particularly with regards to rapidly emerging phenomena, such as police violence. Of course, many of the caveats related to official surveillance systems apply to crowdsourced data, along with the general necessity to assess the validity and reliability of the data collected. Nonetheless, these emergent data systems can produce very useful and timely data under the right circumstances.

Surveillance endeavors can have positive or negative impacts; therefore, a just surveillance system must strive to account for and minimize the possibility that its protocols and mechanisms might inadvertently harm individuals or communities [16]. Such harm occurs when, for instance, the personal information that public health agencies collect about an individual is shared with companies or governmental agencies (e.g., law enforcement) for purposes unrelated to the original public health aims [16].

### 4.2. Implications and Future Directions

The infrastructure of existing public health and other surveillance systems could be used to develop a surveillance system that can track racism, related social stigma and COVID in real time. Given the urgent need for such a system, we offer the following recommendations, which address the collective strengths and weakness of the systems evaluated here.

Draw on the strengths each of the three types of systems has to offer. Doing so provides the best opportunities for developing and sustaining a novel anti-racism COVID monitoring system. This includes the real-time nature of COVID systems, the use of validated measures of psychosocial indicators that are available in other public-health surveillance systems and the inclusion of explicit indicators of racism that key monitoring systems have been using for decades to track racism and racialized stigma.

Consider integrating certain machine learning approaches into traditional survey-based surveillance approaches.

To ensure equity across states, bolster the capacity of state and local health departments to monitor and address the needs of their communities comprehensively. Provide the resources and expertise that STLT health departments need to develop and maintain integrated COVID-19 stigma monitoring systems focused on inequities.

Existing surveillance systems may already include multiple measures of COVID-19-related outcomes, including cases and deaths, as well as hospitalizations and testing. Standardize how key indicators (e.g., COVID-19 test and stigma) are operationalized. This enables comparisons across regions, across populations and over time.

Establish guidelines about which COVID-19-related indicators to include in any system and the best metrics for reporting them. Account for the different ways diverse types of end-users will make use of the information. Absolute numbers can inform decisions regarding the resources needed and the costs of those resources, whereas relative estimates provide insights regarding the epidemiologic significance of the problem, including racial/ethnic inequities.

Include measures of both self-reported race/ethnicity and perceived race/ethnicity, so that end-users can identify disparities and their determinants [35,36]. Whereas the former facilitates administrative tracking in accordance with the US Office of Management and Budget guidelines, the latter serves as an indicator of risk for exposure to certain forms of racism [36,37,38].

Apply a pandemic ethics framework to monitor potential harms and protections of any newly developed system continuously. Communicate this commitment to stakeholders, especially community members. This is important for remaining accountable to community and inviting their involvement in its design [30].

The efforts used to control infectious disease pandemics raise ethical issues [26]. One notable resource, the Pandemic Ethics Dashboard [30], responded to this challenge early in the COVID pandemic by providing guidance to minimize the possibility of COVID-19 mitigation strategies inadvertently harming communities.

However well-intentioned public-health-surveillance efforts may be, it is critical to consider ways they may introduce harm to communities of color through the criminalization of communities that are monitored based in part on their race, gender, class and ability [39]. The alternative model we are pursuing is conducted in partnership with the community, using community engagement and participatory processes. Fortunately, many important collective and community-led organizations are discussing ways to proceed forward and exploring ways to promote data sovereignty, sharing, ownership, transparency and data abolition. These groups are (1) documenting how data and tech are part of a larger surveillance industrial complex that is further disenfranchising and marginalizing communities of color and (2) providing a roadmap on how data and technology can be reimagined for social justice goals.

Surveillance strategies that do not prioritize equity may place socially marginalized populations at elevated risk for inadvertent harm [40]. It is important to establish protocols to ensure system components do not inadvertently harm the very communities they are intended to serve. Historically, public-health surveillance has sought to gather large amounts of information about individuals [41]. Community-informed approaches call for a shift that prioritizes the privacy of individuals and thus focuses on gathering large amounts of information about the inequalities to which they are exposed. Needed still is a way to evaluate the potential harm. We have been developing a scoring system to evaluate these considerations, but we are aware of no system that applies it.

### 4.3. Limitations and Strengths

The primary limitation of this research is that it does not include all possible systems, and given the rapidly evolving nature of the COVID pandemic, the review likely missed some systems. For instance, many systems have emerged or terminated during the pandemic; their inclusion in the baseline assessment would depend in part on the timing during which the system was in place. Strengths of this research include the use of both standard criteria and PHCRP-based criteria to guide the detailed assessments. This approach improves the applicability of the findings for racial health equity efforts.

## 5. Conclusions

This research sought to identify characteristics of existing surveillance systems that might inform the development of a novel COVID monitoring system to track COVID-19-outcomes in real time, while also tracking key forms of stigma and racism that affect them. We conducted a baseline environmental scan of existing COVID and other surveillance systems and drew on content area expertise, feedback from members of an expert panel on surveillance and input from CDC consultants to generate a reduced list for the detailed assessments, which were completed by using standard evaluation criteria, as well as criteria based on PHCRP, an anti-racism research approach rooted in Critical Race Theory. The findings indicate that each type of system offers a different set of strengths that can inform the development of the novel system. The real-time nature of COVID surveillance systems is its greatest strength; overall, however, their approaches to race, ethnicity and racism are inadequate. Other public-health surveillance systems, especially behavioral health systems, offer measures of psychosocial variables and race/ethnicity data that might also be used in the novel system; however, the population-based survey design of these systems contributes to delays in being able to act. Finally, systems that focus on tracking racism provide information that is difficult to find elsewhere; however, the data are often reported on an annual basis, resulting in substantial delays from incident to reporting. These data may also be subject to underreporting bias, because they rely on people to report incidents, which may be difficult for some victims to do. Overall, the key implication is that the novel system should draw on all three types of systems, as their strengths and weaknesses complement one another.

In conclusion, effective surveillance is critical to mitigate public health crises; however, the tools currently available are inadequate to monitor the intersecting crises of racism, related social stigma and COVID. Moreover, the failure to address the urgent need for data on race, ethnicity, health inequities and racism may constitute a form of institutionalized racism that reinforces White supremacy by hampering the development of evidence-based practice and policy to address the inequities [2,42]. This evaluation of existing resources can inform the development of new surveillance tools that are rooted in equity, that target racism directly and that consider the inadvertent ways surveillance has harmed the racialized and marginalized communities that public-health efforts are intended to help.

## Figures and Tables

**Table 1:** Criteria guiding the evaluation of existing systems.

Criteria	Key Considerations
**For Surveillance in General**	
Usefulness	To what extent does the system support the achievement of the stated goals?
Timeliness	How long does it take for the system to acquire needed data and make them available?
Flexibility	To what extent can the system adapt to new circumstances or needs?
Simplicity	How easy is it for users to operate the system?
Data Quality *	How complete and accurate are the data fields in the reports the system receives? How reliable are the data?
**Informed by Public Health Critical Race Praxis**	
Race/ethnicity data	Which race/ethnicity data are included? To what extent do they support the achievement of Project REFOCUS aims?
Stigma measures	What valid measures of stigma are included?
Racism measures	What relevant measures of racism are included?
Surveillance Implications	To what extent might the system contribute to harm of racial/ethnic minority and vulnerable populations or aid community-originated surveillance projects?

* Where this information is available.

**Table 2:** Key characteristics of selected COVID-19 surveillance systems.

	COVID-19 Outcomes ^a^						Measures	Race/Ethnicity ^b^									Updates
System Name	T	C	H	V	D	O	Stigma	Racism	W	B	L	A	NA/AN	H/PI	O	N/A	
1point3acres	x	x			x	x			x	x	x	x	x	x			Daily
Olivia	x	x		--	x	--	--	--	--	--	--	--	--	--	--	x	Weekly
JHU COVID-19 Dashboard	x	x			x	x	--	--	--	--	--	--	--	--	--	x	Daily
COVID-19 Case Surveillance	?	x	x	?	x	x	--	--	x	x	x	x	x	x	x	x	Daily
COVID Tracking Project	x	x	x	--	x	--	--	--	x	x	x	x	x	x	x	x	Daily
LA County Dept. of Public Health	x	x	x	x	x	x	--	--	x	x	x	x	x	x	x	x	Daily
COVID Behind Bars project	x	x			x	x	--	--	--	--	--	--	--	--	--	--	Daily
CDC COVID Data Tracker		x		--	x	x	--	--	x	x	x	x	x	x	x	x	Daily
NC DHHS COVID-19 Response	x	x	x		x	x			x	x	x	x	x	x	x		Weekly
Census COVID Data	x	x	x	x	x	x	x	x	x	x	x	x	x	x	x	x	?
Google COVID-19 Public Forecaster	?	x	?	?	x	x	--	--	?	?	?	?	?	?	?	?	Continuous
National Vital Statistics Program					x	x			x	x		x	x	x		x	4 November

^a^ T = test (viral or antibody), C = cases (i.e., diagnoses), H = hospitalizations, V = ventilators used, D = deaths; O = Other/do not know. ^b^ Race/ethnicity categories: W = White; B = Black, L = Latino; A = Asian; NA/AN = Native American Alaska Native; H/PI = Hawaiian or other Pacific Islander; O = other race; DK = Do not know.

**Table 3:** Key characteristics of other public-health surveillance systems.

System	Race/Ethnicity ^a^								Measures		COVID-19 Outcomes ^b^						
	W	B	L	A	NA/AN	H/PI	O	DK	Stigma	Racism	T	C	H	V	D	O	Updates
National Longitudinal Study of Adolescent to Adult Health	x	x	x	x	x	x	x	x	x	x	--	--	--	--	--	--	Annual
Amerispeak/NORC General Social Survey	x	x	x	x	?	?	?	x	x	x	--	--	--	--	--	x	Biennial
California Health Interview Survey	x	x	x	x	x	x	x	x	x	x	x	x	x	x	x	x	Monthly, Annual
CDC BRFSS	x	x	x	?	?	?	x		x	x	--	--	--	--	--		Annual
CDC Influenza Surveys	--	--	--	--	--	--	--	x	--	--	x	x	?	--	x	x	Varies
CDC INFO Query	x	x	x	x	x	x	x		x *	x	--	--	--	--	--	x	Continuous
Current Population Survey	x	x	x	x	x	x	x	x	x	x	--	x	--	--	x	--	Monthly, Annual
National Syndromic Surveillance Program	x	x	x	x	x	x	x	x			x	x	x	x	x	x	Continuous
Youth Risk Behavior Surveillance Survey	x	x	x	x	x	x			x		x	x	x	x	x	x	Varies, Continuous

^a^ Race/ethnicity categories: W = White; B = Black, L = Latino; A = Asian; NA/AN = Native American Alaska Native; H/PI = Hawaiian or other Pacific Islander; O = other race; DK = Do not know. ^b^ T = test (viral or antibody), C = cases (i.e., diagnoses), H = hospitalizations, V = ventilators used, D = deaths; O = Other/do not know. * If this information is reported.

**Table 4:** Key characteristics of racism and racialized-stigma monitoring systems.

System Name	Updates	COVID-19 Outcomes ^a^ [1]						Race/Ethnicity Data ^b^								Key Measures	
		T	C	H	V	D	O/DK	W	B	L	A	NA/AN	H/PI	O	N/DK	Stigma	Racism
Decennial Census	Decade	x	x	x	x	x	x	x	x	x	x	x	x	x			x
American Community Survey	Annual							x	x	x	x	x	x	x			x
Home Mortgage Disclosure Act	Varies, Quarterly, Annual							x	x	x	x	x	x	x			x
Project Implicit	n/a							x	x	x	x	x				x	x
Twitter	Continuous	x	x	?	?	x	x									x	x
Google	Continuous						x									x	x
FBI Hate Crimes	Annual							x	x	x	x	x	x	x	x		x
Equal Opportunity Employment Commission	Annual							x	x	x	x	x	x				x
Pew Research Center	Varies							x	x	x	x			x	x	x	x
NORC General Social Survey	Varies							x	x	x				x		x	x
STOP AAPI Hate	Continuous										x		x			x	x

^a^ T = test (viral or antibody), C = cases (i.e., diagnoses), H = hospitalizations, V = ventilators used, D = deaths; O = Other/do not know. ^b^ Race/ethnicity categories: W = White; B = Black, L = Latino; A = Asian; NA/AN = Native American Alaska Native; H/PI = Hawaiian or other Pacific Islander; O = other race; DK = Do not know.
